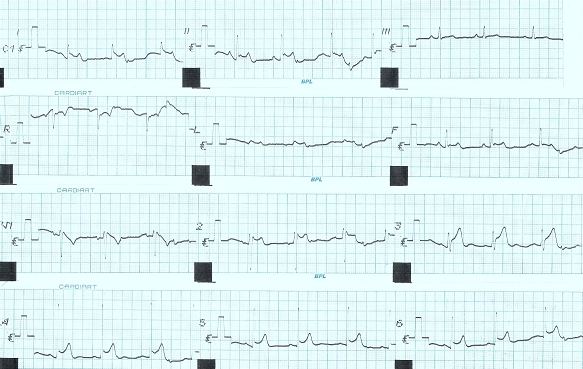# Brugada-type Electrocardiographic Pattern Induced by Electrocution

**Published:** 2009-01-07

**Authors:** R Rangaraj, Nagaraja Moorthy, Shivanand S Patil, CN Manjunath

**Affiliations:** Department of cardiology, Sri Jayadeva Institute of Cardiology, Bangalore, India

**Keywords:** Electrocution, Brugada syndrome, early repolarisation, ventricular fibrillation, sudden cardiac death

## Abstract

Heart is one of the most frequently affected organs in electrocution. Electrical injury can cause life-threatening cardiac complications such as asystole, ventricular fibrillation, and myocardial rupture. In this case report, we describe a 22-yr-old male patient who sustained electric burn injury and presented with electrocardiogram showing transient Brugada type pattern.

## Introduction

Electrical injury can cause life threatening cardiac arrhythmias, myocardial/valvular rupture, structural changes in coronary arteries, pericardial effusion, and various electrocardiographic changes. The Brugada syndrome is a distinct type of the idiopathic ventricular fibrillation, which is characterized by ST-segment elevation in right precordial leads (V1-V3), right bundle branch block (RBBB) pattern, and high incidence of sudden cardiac death (SCD) in patients with structurally normal hearts [1]. In this report, we describe a patient who presented with transient Brugada type electrocardiographic pattern with early repolarisation following electrocution.

## Case Report

A 22-yr-old male, farmer by occupation was referred to our tertiary cardiac care institute for management of abnormal ECG changes mimicking acute coronary syndrome. He had sustained accidental electric burn injury (240V) while carrying out some work at home 2 hours prior to admission. He was thrown to the ground following an electrocution and was found to have brief period of loss of consciousness for few seconds with spontaneous recovery. He was immediately attended at a local hospital where ECG changes were noticed  and referred to our institution for opinion and further management. He had no prior history of cardiac illness. There was no history of chest pain, palpitation or neurological deficits. There was no history suggestive of sudden or premature cardiac death in his family. He was not on any medications.

On physical examination, patient was conscious alert and had deep electric burn entry wound over the dorsum of left hand and an exit wound over the left ankle. The arterial blood pressure and heart rate were 120/80 mmHg and 78 beats per minute, respectively. Cardiovascular assessment was normal. Other systemic examinations were normal. Surface electrocardiogram done immediately following injury demonstrated a sinus rhythm with RBBB with coved ST segment elevation with negative T wave in V1 mimicking type 1 Brugada pattern and saddle back ST segment with upright T wave in V2 mimicking type 2 Brugada pattern with early repolarisation (Figure 1). QTc was normal. Echocardiographic assessment revealed a normal left ventricular size and function, and no valvular pathology. Routine laboratory tests, including blood urea, serum creatinine, serum electrolytes and cardiac biomarkers were normal. Repeat ECG at 2 hours showed disappearance of above mentioned Brugada type pattern with persistent early repolarisation. Patient was observed in the ICU for rhythm disturbances for 24 hours. Repeat ECG after 24 hours was normal   pattern. Since patient had no family history of sudden cardiac death and family members showing normal ECG and clear precipitating event that can result in above mentioned ECG changes he was not subjected for further investigations like induction with class I antiarrhythmics or electrophysiological studies.

## Discussion

Heart is one of the most vulnerable organs against electricity. Cardiac effects of electrical shock can be divided into arrhythmias, conduction abnormalities, and myocardial damage. Various myocardial manifestations develop at the time of injury. These include asystole, ventricular fibrillation, which may cause immediate death, QT-prolongation, right bundle branch block, complete AV block, valvular or myocardial rupture, CK-MB elevations caused by myocardial injury, structural changes in the small coronary vessels, and pericardial effusion [2]. Damage to the myocardium may occur after exposure to high- and low-voltage current. Injury is caused directly by electrothermal conversion and electroporation or secondarily by contusion following a lightning strike. Other described mechanisms include coronary spasm leading to ischemia and arrhythmias inducing hypotension and secondary coronary hypoperfusion [3,4].

Sudden cardiac death due to ventricular fibrillation is more common with low-voltage alternating current, whereas asystole is more frequent with electric shocks from direct current or high-voltage alternating current. Incidence of ventricular fibrillation was inversely proportional to voltage and the occurrence of ventricular tachycardia and atrial fibrillation were directly proportional to voltage [5]. The most common arrhythmias are sinus tachycardia and premature ventricular contractions, but ventricular tachycardia and atrial fibrillation have been reported. Most arrhythmias occur soon after the electrical shock, but delayed ventricular arrhythmias (noted up to 12 hours following an incident) may occur [6]. Most patients not experiencing sudden cardiac death have nonspecific ST–T-wave abnormalities on 12-lead electrocardiography (ECG) that usually resolve spontaneously. Patients without ECG changes on presentation are unlikely to experience life-threatening arrhythmias [7].

The Brugada syndrome is characterised by a distinct type of ST-segment elevation in right precordial leads (V1-V3), right bundle branch block (RBBB) pattern, and high incidence of sudden cardiac death (SCD) in patients with structurally normal hearts [1]. The diagnosis of the syndrome is easily obtained by electrocardiography as long as the patient presents the typical ECG pattern and there is a history of aborted sudden death or syncopes caused by a polymorphic VT.

The typical ECG findings are conduction delay and/or premature repolarisation in the right precordial leads V1-V3. The ST segment is downsloping with an inverted T wave. This is often described as 'coved' type ST shift. The widened S waves characteristic of true RBBB are absent in the lateral leads. The T wave may also be positive giving the 'saddleback' ST elevation. The ECG may change from 'coved' to 'saddle back' and may even normalise over time in approximately 40% of cases [8]. Depression or loss of the action potential dome in RV epicardium creates a transmural voltage gradient that may be responsible for the ST-segment elevation observed in the Brugada syndrome and other syndromes exhibiting similar ECG manifestations [9].

Brugada syndrome is thought to be primarily a disease of cardiac conduction and it has been linked to abnormalities in the sodium channel (SCN5A). These patients present with sudden cardiac death at rest or in their sleep and have Brugada shift on their ECG. The rest of their history and physical examination are unremarkable. A family history of sudden death, syncope or ventricular fibrillation is present in 22% [10]. Electrical heterogeneity within right ventricular epicardium leads to the development of closely coupled extrasystoles via a phase 2 reentrant mechanism, which then precipitate ventricular tachycardia-ventricular fibrillation. The incidence of sudden death in this syndrome is very high and, at present, implanting a cardioverter defibrillator can only prevent sudden death.

However our patient did not have chest pain or showed no elevation in the cardiac biomarkers or wall motion abnormality on echocardiogram. So the ECG changes mimicking Brugada pattern could be explained with the electrical injury of myocardium leading to spatial dispersion of repolarisation.

Probably in our patient the electrocution has precipitated transient Brugada type electrocardiographic pattern that became normal over few hours and the syncopal attack could be due to non-sustained ventricular arrhythmias. Thus we postulate that the higher incidence of ventricular arrhythmias and sudden death in patients with electrocution could be due to Brugada like electrocardiographic changes of repolarisation. We also recommend that emergency care physicians should be aware of these electrocardiographic changes and these patients need close monitoring of rhythm to prevent sudden cardiac death.

## Conclusion

In conclusion, electrical injury can cause transient Brugada type electrocardiographic pattern. Such patients may be at high risk of developing life threatening ventricular arrhythmias and sudden death. Patients with electrocution induced Brugada type electrocardiographic pattern need close monitoring for life threatening cardiac arrhythmias.

## Figures and Tables

**Figure 1:**